# Interference activity of a minimal Type I CRISPR–Cas system from *Shewanella putrefaciens*

**DOI:** 10.1093/nar/gkv882

**Published:** 2015-10-10

**Authors:** Srivatsa Dwarakanath, Susanne Brenzinger, Daniel Gleditzsch, André Plagens, Andreas Klingl, Kai Thormann, Lennart Randau

**Affiliations:** 1 Prokaryotic Small RNA Biology, Max Planck Institute for Terrestrial Microbiology, Marburg, Hessen D-35043, Germany; 2 Institute for Microbiology and Molecular Biology, Justus-Liebig-University Giessen, Giessen, Hessen D-35392, Germany; 3 Plant Development, Department Biology I, Biocentre LMU Munich, Großhaderner Str. 2–4, Planegg-Martinsried D-82152, Germany; 4 LOEWE Center for Synthetic Microbiology (Synmikro), Marburg, Hessen D-35043, Germany

## Abstract

Type I CRISPR (Clustered Regularly Interspaced Short Palindromic Repeats)–Cas (CRISPR-associated) systems exist in bacterial and archaeal organisms and provide immunity against foreign DNA. The Cas protein content of the DNA interference complexes (termed Cascade) varies between different CRISPR-Cas subtypes. A minimal variant of the Type I-F system was identified in proteobacterial species including *Shewanella putrefaciens* CN-32. This variant lacks a large subunit (Csy1), Csy2 and Csy3 and contains two unclassified *cas* genes. The genome of *S. putrefaciens* CN-32 contains only five Cas proteins (Cas1, Cas3, Cas6f, Cas1821 and Cas1822) and a single CRISPR array with 81 spacers. RNA-Seq analyses revealed the transcription of this array and the maturation of crRNAs (CRISPR RNAs). Interference assays based on plasmid conjugation demonstrated that this CRISPR-Cas system is active *in vivo* and that activity is dependent on the recognition of the dinucleotide GG PAM (Protospacer Adjacent Motif) sequence and crRNA abundance. The deletion of *cas1821* and *cas1822* reduced the cellular crRNA pool. Recombinant Cas1821 was shown to form helical filaments bound to RNA molecules, which suggests its role as the Cascade backbone protein. A Cascade complex was isolated which contained multiple Cas1821 copies, Cas1822, Cas6f and mature crRNAs.

## INTRODUCTION

The arms race between viruses on one end and bacteria and archaea on the other end resulted in the evolution of diversified prokaryotic immune systems and viral countermeasures (1,2). Prokaryotic genomes can contain adaptive immune systems, termed CRISPR (Clustered Regularly Interspaced Short Palindromic Repeats)–Cas (CRISPR-associated) (3,4), which utilize small RNAs to target foreign genetic elements (5,6). Genomic CRISPR elements consist of an array of short repeat sequences that are interspersed with unique spacer sequences that can be derived from viral genomes and conjugative plasmids (7–9). CRISPR arrays are often located adjacent to a set of *cas* genes that are essential for conferring immunity (10,11). The CRISPR–Cas immune response can be divided into three stages. First, upon infection of the host by a virus, a segment of a viral genome, i.e. the protospacer, is integrated as a new spacer into an expanding CRISPR array (12). This stage of the immune response, termed acquisition, relies on the recognition of a short signature sequence, the protospacer adjacent motif (PAM) in the viral DNA sequence (13,14). Next, the CRISPR array is transcribed and processed into short CRISPR RNAs (crRNAs) that contain a spacer sequence flanked by parts of the repeats at their termini (15–19). The crRNAs are integrated into CRISPR ribonucleoprotein (crRNP) surveillance complexes that are formed by multiple Cas proteins. These complexes utilize the crRNA guidance to recognize and degrade foreign genetic material during a recurring infection (6,17,20–22). The recognition of the foreign genetic material is achieved via Watson–Crick base pairing between the crRNA and the unwound target DNA strand or target RNA sequence (23–25).

CRISPR–Cas systems are classified into three main types based on the presence of the signature proteins Cas3 (Type I), Cas9 (Type II) and Cas10 (Type III), respectively (10,26). Only Cas1 and Cas2 are conserved in the vast majority of the CRISPR–Cas systems and have been found to be essential for the acquisition of new spacers (13,27,28). Type I systems utilize a crRNP complex termed Cascade (or CRISPR-associated complex for antiviral defense) to identify targets (6,21,29), and Type II systems are characterized by the standalone nuclease Cas9 (30–32). In both cases, the recognition of targets is dependent on the presence of a PAM sequence (25,33,34). In contrast, Type III systems use crRNP complexes that facilitate co-transcriptional ssRNA and DNA cleavage in a PAM sequence-independent manner (20,35,36).

The Type I Cascade crRNP complex has been studied biochemically and structurally for subtype I-E (17,23,24), and cryo-EM structures are available for Cascade complexes of subtypes I-A, I-C and I-F (37–39). The 405 kDa Type I-E Cascade from *Escherichia coli* has a characteristic ‘seahorse-shape’ architecture and is formed by Cas5e, Cas6e, Cas7 and two subtype-specific proteins Cse1 (large subunit) and Cse2 (small subunit) (23,24,37). The complex has an uneven protein stoichiometry of (Cas7)_6_–(Cse2)_2_–(Cse1)_1_–(Cas5e)_1_–(Cas6e)_1_ and binds to a 61 nt-long crRNA, which contains a 32 nt-long spacer flanked by 8 nt- and 21 nt-long repeat sequences, respectively. Cse1, the large subunit protein of the Cascade complex, is involved in PAM recognition and recruits Cas3 after target recognition (17,37,40), whereas the small subunit protein Cse2 functions in stabilizing the R-loop by binding to the displaced DNA strand (17,23,24). The Type I-E Cascade has a helical backbone formed by six copies of Cas7 and contains a groove in which the crRNA is bound and protected. Additionally, the 3′- and 5′-ends of the crRNA are protected by Cas6e and Cas5e, respectively (17,41). This general architecture of the Cascade crRNPs appears to be conserved for all Type I subtypes, even though their Cas protein compositions can differ considerably (42).

One apparent variation of this Cascade architecture is evident for a computationally identified subtype I-F variant present in few beta- and gammaproteobacteria. This variant was first described for *Photobacterium profundum* SS9 (PBPRB1995-PBPRB1991) and was suggested to rely on a minimal set of five Cas proteins (26). The system contains Cas1, an integrase that mediates spacer acquisition (43,44), Cas2–3, the target DNA nuclease, Cas6f, the endonuclease that generates mature crRNAs (18), and two additional genes that show no apparent sequence similarity to any known Cas protein families. Furthermore, a large subunit could not be detected (26). We identified this minimal CRISPR-Cas subtype in the *Shewanella putrefaciens* strain CN-32 and experimentally verified crRNA maturation and DNA interference activity *in vivo*. Recombinant production of the Cas proteins revealed the presence of a Cascade complex and allowed for the assignment of one unclassified Cas protein into the Cas7 group. The two unclassified proteins were shown to be required for a stable crRNA pool in *S. putrefaciens* CN-32. Our results provide insights into the reductive evolution of CRISPR–Cas systems and demonstrate that a Cascade complex can function without a large subunit.

## MATERIALS AND METHODS

### Bacterial strains and growth conditions

Bacterial strains and plasmids used in this study are listed in Supplementary Table S1. *E. coli* strains DH5α, DH5α λpir and WM3064 were grown in LB medium at 37°C. Media for the growth of the 2,6-diamino-pimelic acid (DAP)-auxotroph *E. coli* WM3064 strain were supplemented with 300 μM of DAP. *E. coli* strain BL21(DE3) pLysS was grown either in LB or in NZ-amine (1% NZ-amine, 0.5% yeast extract and 1% NaCl) media at 37°C until an OD_600_ of ∼0.6 was reached. Protein expression was induced by addition of 1 mM Isopropyl β-d-thiogalactopyranoside (IPTG) and continuous growth at 18°C. *S. putrefaciens* CN-32 was cultured in LB medium at 30°C. Media were supplemented with 50 μg/ml spectinomycin, 50 μg/ml kanamycin, 50 μg/ml ampicillin or 10% (w/v) sucrose.

### Construction of *cas* gene deletion strains

*S. putrefaciens* CN-32 mutants with markerless in-frame deletions or integrations were constructed using the primers listed in Supplementary Table S2 and the suicide vector pNPTS138-R6KT (Kan^R^) following the procedure reported in (45). Briefly, appropriate ∼500 bp homologous fragments of the up- and downstream regions of the target gene were amplified. For deletions, the codons encoding the first and last four amino acids were retained. The resulting PCR-derived DNA fragments were joined via overlap PCR, treated with appropriate restriction enzymes and ligated with the equally digested pNPTS138-R6KT vector. The resulting plasmids were sequenced and transferred to *S. putrefaciens* CN-32 by conjugation from *E. coli* WM3064. Target gene regions were replaced by the corresponding modified version via sequential homologous crossover and correct deletions were identified via colony PCR using appropriate primers.

Point mutations were introduced into the *S. putrefaciens* CN-32 gene *Sputcn32_1822*. To this end, the wild-type gene sequence, along with 500 bp of upstream and downstream sequences was cloned into pNPTS138-R6KT. Point mutations were introduced into this plasmid by QuickChange site-directed mutagenesis (Stratagene) using primers that were designed using the Agilent QuickChange Primer Design Tool. The plasmids carrying the point mutations were applied for in-frame insertions in *S. putrefaciens* CN-32 as described above.

### RNA isolation, sequencing and Northern blot analysis

Nucleic acids were extracted from an overnight culture of *S. putrefaciens* CN-32 using phenol/chloroform (1:1 phenol pH 5 for RNA and pH 8 for DNA). The mirVana RNA extraction kit (Ambion) was used to isolate small RNAs (<200 nt) from total RNA. To ensure proper termini for adapter ligation, 1 μg of the small RNA preparation was incubated with 10 U of T4 polynucleotide kinase (T4 PNK, Ambion) at 37°C overnight, followed by 1 h incubation at 37°C in presence of 1 mM ATP. This treatment ensures 5′-monophosphate termini suitable for RNA-Seq library preparations. RNA libraries were sequenced by Illumina HiSeq2000 sequencing at the Max-Planck Genome Centre, Cologne.

Northern blot analyses required the extraction of 10 μg of total RNA from wild-type and *cas* gene-knockout strains of *S. putrefaciens* CN-32. The RNA preparations were denatured (95°C for 5 min) in formamide loading buffer and separated via electrophoresis on a 10% TBE–8 M urea polyacrylamide gels at 200 V for 1 h. The separated bands were transferred to a nylon membrane (Roth) and subjected to UV-crosslinking at 25 V for 2 h. DNA probes were radiolabeled with 5′-γ[^32^P]-ATP using T4 PNK and incubated with the membrane, which was pre-incubated at 42°C for 30 min with ULTRAhyb-Oligo buffer (Ambion). After overnight hybridization at 42°C, the membranes were washed two times for 15 min with 2× SSC buffer and 0.1% SDS and with 0.1× SSC and 0.1% SDS. Radioactive signals were detected by phosphorimaging.

### Conjugation-assays for DNA interference analysis

Target plasmids for conjugation assays were designed with the primers listed in Supplementary Table S2. Individual primer pairs were hybridized generating the sticky ends of EcoRI and BamHI restriction sites that flanked the protospacer sequences. The primer pairs were phosphorylated at the 5′-termini by T4 PNK, mixed and hybridized (95°C for 5 min, followed by slow cooling to room temperature). The obtained DNA fragments were ligated into a linearized pBBR1MCS2 (Kan^R^) vector. Insertion of the fragment was confirmed via sequencing.

The obtained plasmids were transformed into the donor strain *E. coli* WM3064. Equal amounts of *E. coli* and *S. putrefaciens* CN-32 strains overnight cultures were harvested. The strains were suspended in 100 μl of DAP-supplemented LB medium and mixed before spotting a single drop on nonselective LB agar supplemented with DAP. After overnight incubation at 30°C, the cells were washed from the plate using 2 ml of nonselective LB. The mating mixture was then washed three times with 1 ml of nonselective LB and screened for conjugated *S. putrefaciens* CN-32 by plating 100 μl of the mating mixture on LB plates supplemented with Kan but lacking DAP. This procedure was performed in triplicate and in parallel for the control plasmid without protospacer sequences. The number of obtained colonies was counted for conjugation of the plasmid with protospacer (pT) and the control plasmid (pNT). Relative conjugation efficiency was calculated as pT/pNT ratio and the transconjugant counts are detailed in Supplementary Table S3.

### Production and purification of recombinant proteins

The *cas* genes of *S. putrefaciens* CN-32 were amplified from genomic DNA and cloned into gene expression vectors. The *cas6f* gene was cloned into pET20b (Amp^R^) to generate Cas6f with a C-terminal His-tag. The gene cassette *cas1821, cas1822, cas6f* was cloned into pRSFDuet; this construct allows the simultaneous production of all three proteins with only Cas1821 having an N-terminal His-tag fusion. The individual *cas1821* gene was cloned to produce a His-Sumo-tag fusion that was processed as previously described for *Thermoproteus tenax* Cas proteins (22). Cas6f production was induced by addition of 1 mM IPTG followed by overnight growth at 18°C of the *E. coli* BL21(DE3)pLysS host strain. Cell lysates were pepared by sonication in a buffer containing 50 mM Tris-HCl pH 8.0, 500 mM NaCl, 5% glycerol, 1 mM dithiothreitol (DTT), 0.01% Triton X100 and 20 mM imidazole. Proteins were purified on a HiTrap Ni-NTA column (GE Healthcare) in a linear imidazole gradient ranging from 100 to 500 mM imidazole. The Cas1821 protein was subjected to Ni-NTA chromatography in a buffer containing 100 mM potassium phosphate pH 7.5, 500 mM NaCl, 10% glycerol. A high-salt washing step with a buffer including 1 M NaCl was added before protein elution to remove nucleic acid contaminants. The SUMO tag was cleaved using SUMO protease during overnight dialysis at 4°C in a buffer containing 50 mM Tris–HCl pH 7.0, 100 mM NaCl and 10% glycerol. The protein was further purified over a HiTrap Heparin Sepharose HP column and size exclusion chromatography on a Superdex 200 column.

The production of recombinant Cascade required the presence of the pRSFDuet vector containing the genes *cas1821, cas1822, cas6f* and a pUC19 vector containing the repeat-spacer4-repeat sequence from the CRISPR array cloned downstream of a T7 RNA polymerase promoter. The 100 nt transcript is processed by Cas6f into a 60 nt crRNA. BL21(DE3)pLysS cells with both plasmids were grown in the presence of ampicillin, kanamycin and spectinomycin. Protein and pre-crRNA production was induced at an OD_600_ of 0.6 by addition of 1 mM IPTG, followed by overnight growth at 18°C. Cascade crRNP complexes were purified via Ni-NTA chromatography in a buffer containing 50 mM Tris-HCl pH 7.0, 500 mM NaCl, 10% glycerol, 1 mM DTT and 20 to 500 mM imidazole. Size exclusion chromatography was performed on a Superdex 200 column (calibrated with a molecular weight standard kit (12 000–200 000 Da, Sigma-Aldrich)) in a buffer containing 50 mM HEPES-NaOH pH 7.0, 150 mM NaCl and 1 mM DTT.

### Transmission electron microscopy of Cas1821

Purified protein samples were negatively stained with 2% (w/v) uranyl acetate as described previously (22,46,47). Subsequent electron microscopy was carried out with a JEOL JEM-2100 transmission electron microscope equipped with a LaB_6_ cathode at 120 kV (JEOL, Tokyo, Japan). Electron micrographs were taken with a 2k × 2k fast-scan CCD camera F214 combined with EM-Menu 4 (TVIPS, Gauting, Germany).

## RESULTS

### Identification of a minimal CRISPR-Cas subtype in *S. putrefaciens* CN-32

**Figure 1.**
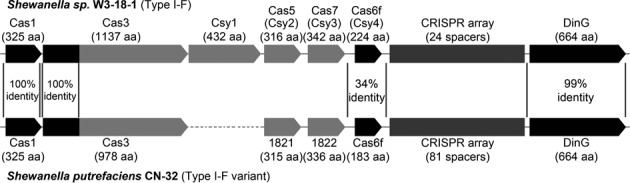
Comparison of CRISPR-Cas systems in *S. putrefaciens* strains. Schematic diagrams show the single CRISPR-Cas systems from *S. putrefaciens* W3–18–1 (Sputw3181_2191- Sputw3181_2185, Type I-F) and *S. putrefaciens* CN-32 (Sputcn32_1819 – Sputcn32_1824, Type I-F variant). The N-terminal 94 amino acids of the Cas3 proteins share 100% aa identity. The C-terminal Cas3 portion and the Cascade components do not reveal significant protein similarity.

A minimal Type I-F variant CRISPR–Cas subtype was first described for *Photobacterium profundum* (PBPRB1995-PBPRB1991) (26). The activity of this unusual system has not been evaluated experimentally. We identified a similar system in *S. putrefaciens* CN-32 (Figure 1). *S. putrefaciens* CN-32 harbors a single CRISPR array with 81 spacers and only five *cas* genes: *cas1* (Sputcn32_1819), *cas3* (Sputcn32_1820), *cas6f* (Sputcn32_1823), *cas1821* and *cas1822*. The comparison of this system with the subtype I-F system present in the *Shewanella putrefaciens* strain W3–18–1 illustrates the loss of the large subunit and the possible diversification of the Csy2 and Csy3 proteins (Figure 1A). These CRISPR-Cas systems share 99–100% amino acid identity for the flanking Cas1 and a CRISPR-array adjacent DinG-helicase. In addition, the Cas6f (Csy4) endonuclease and the N-terminal Cas2-like domain of the Cas2–3 nuclease show significant sequence identity. Homology searches of Cas1821 and Cas1822 revealed the presence of similar I-F variant systems in few other beta- and gammaproteobacteria. These include the human pathogens *Legionella pneumophila* (strain 2300/99 Alcoy) and *Vibrio cholerae* (strain TM 11079–80).

### The CRISPR array in *S. putrefaciens* CN-32 is transcribed and processed *in vivo*

**Figure 2.**
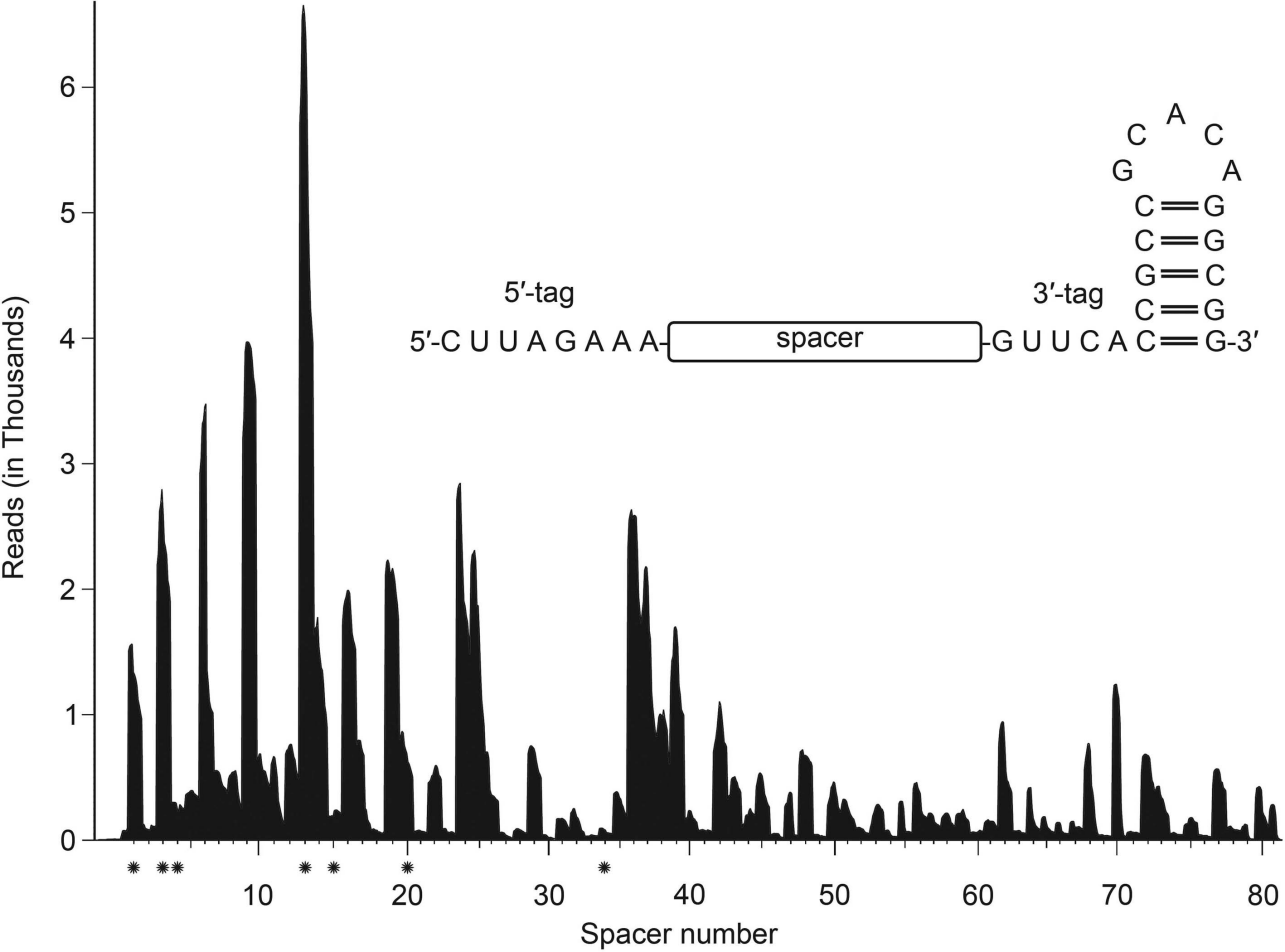
Analysis of CRISPR RNA abundance and termini. The coverage plot of *S. putrefaciens* CN-32 sequence reads (Illumina HiSeq2000) indicates the variable abundance of individual crRNAs. Mature crRNAs contained 5′-terminal 8 nt tags (5′-CUUAGAAA-3′) and 20 nt 3′-ends without further trimming events (inset). Spacers that are marked with an asterisk were tested for interference activity (see Figure 4)

First, we aimed to show crRNA production of the minimal Type I-F variant system. The small RNA fraction was isolated from *S. putrefaciens* CN-32 and subjected to RNA-Seq analyses. Sequencing of crRNAs required T4 PNK treatment of the isolated small RNA molecules during Illumina Hiseq2000 library preparation which indicates the presence of crRNA 5′ OH termini. More than 11 million sequence reads were mapped to the *S. putrefaciens* reference genome. The presence of all 81 crRNA transcripts could be verified and precursor transcript processing generated the expected 8 nt repeat tags at the crRNAs’ 5′-ends (5′-CUUAGAAA-3′) and a 20 nt repeat tag at the 3′-termini (Figure 2). 73 377 reads mapped to the CRISPR locus, indicating a lower crRNA abundance in comparison to very similar RNA-Seq studies for type I-A and type I-B systems (16,22). A minimum free-energy five base pair stem-loop was predicted in the 3′-repeat tags of all crRNAs, which could explain the absence of additional 3′-terminal degradation for most crRNAs (Figure 2). The abundance of the individual crRNAs varied significantly.

We verified that the crRNAs are matured by Cas6f. *Sputcn32_1823* encodes a protein with 37% amino acid identity to Csy4 (Cas6f) from *Pseudomonas aeruginosa*. A sequence alignment of the two proteins verified a conserved histidine residue at position 29 that was previously reported to be essential for Cas6f activity (18). The purified recombinant *S. putrefaciens* Cas6f protein showed endonuclease activity and cleaved a 100 nt-long spacer-repeat–spacer transcript. This endonuclease activity was dependent on the catalytic His29 residue, and a His29Ala point mutation abolished the activity (Supplementary Figure S1).

### The *S. putrefaciens* CRISPR–Cas systems shows *in vivo* interference activity

**Figure 3.**
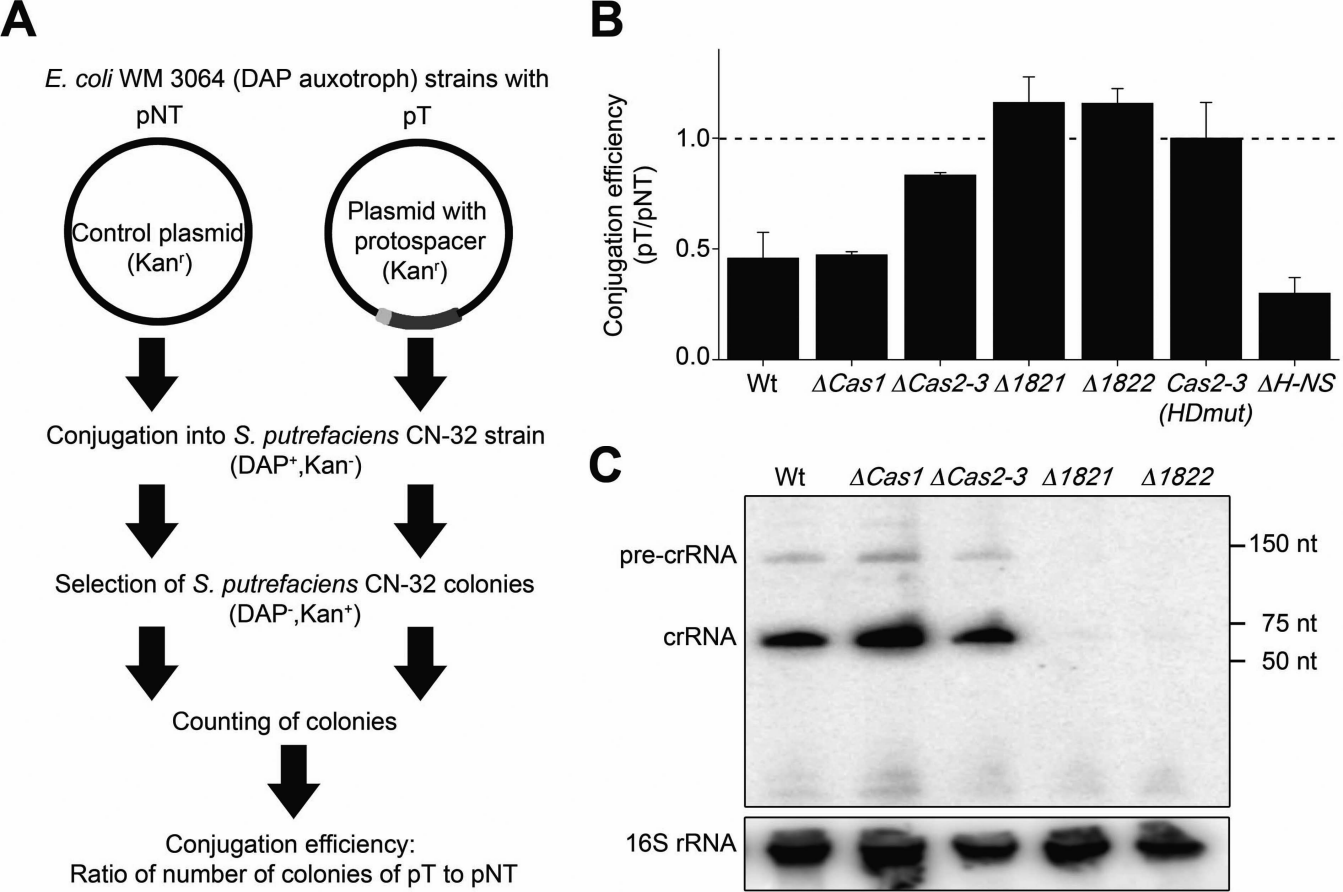
Conjugation assays reveal DNA interference activity of the *S. putrefaciens* CN-32 CRISPR–Cas system. (**A**) The experimental set-up of the conjugation assay is depicted. Two plasmids were used: (I) a control plasmid (pNT, plasmid non-target) and (II) a plasmid containing a PAM sequence (light grey) and a sequence matching spacer 13 (dark grey) of the *S. putrefaciens* CN-32 CRISPR array (pT, plasmid target). The number of *S. putrefaciens* CN-32 colonies carrying each plasmid was counted and the conjugation efficiency was determined. Interference is observed if pT/pNT is lower than 1 in triplicate assays. (**B**) The pT/pNT ratio is calculated for conjugation assays into *S. putrefaciens* CN-32 wild type cells and strains containing deletions of genes encoding Cas1, Cas3, Cas1821, Cas1822 and H-NS. Additionally, a Cas3 active site variant (HDmut, H156A/D157A) was tested. (**C**) Northern blot analyses were performed with extracted total RNA from *S. putrefaciens* CN-32 wild-type and *cas* gene-knockout strains using a 5′-γ-[^32^P]-ATP labeled probe complementary to the repeat sequence. A stable crRNA pool was absent in the *S. putrefaciens* CN-32 Δ*cas1821* and Δ*cas1822* strains.

Next, we assayed CRISPR-Cas-mediated DNA interference activity in *S. putrefaciens* CN-32. The low abundance of crRNAs required the establishment of a sensitive conjugation-based *in vivo* assay (Figure 3A). Two conjugative plasmids were used: (i) a non-target control plasmid (pNT = pBBR1MCS2) and (ii) a target plasmid (pT) containing the 13th spacer of the *S. putrefaciens* CN-32 CRISPR array (spacer13) and the PAM sequence ‘GG’ at the 3′ end of the protospacer. The PAM sequence was selected based on computational predictions by Mojica *et al*. (14). In agreement, we could identify potential prophage targets for spacer33 (∼97% identity between spacer and target) and spacer34 (100% identity between spacer and target) using the tool CRISPRtarget (48). In both cases, the di-nucleotide GG was found at the 3′ end of target strand match. The two plasmids pNT and pT were conjugated from *E. coli* WM 3064 into *S. putrefaciens* CN-32. In addition, markerless gene deletions were constructed for the genes *cas1, cas3, cas1821* and *cas1822* and the two plasmids were conjugated into these deletion strains. The colonies carrying pNT or pT were counted and the relative conjugation efficiency was calculated as pT over pNT. Relative conjugation efficiency (pT/pNT) below 1 indicates interference activity. Interference was observed in wild-type *S. putrefaciens* CN-32 cells (Figure 3B) and the Δ*cas1* strain, which is in agreement with an exclusive role of Cas1 in spacer acquisition. In contrast, interference activity was not apparent in the Δ*cas1821* and Δ*cas1822* strains, and was significantly reduced in the Δ*cas3* strain and a strain carrying a Cas3 mutant that lacks the HD nuclease motif. To investigate the reason for this loss of interference activity, we assayed the crRNA pool in these deletion strains. Total RNA was isolated from the different *S. putrefaciens* CN-32 cells and Northern blot analyses were performed using a radioactively labeled probe against the crRNA repeat sequence (Figure 3C). The production of crRNAs was observed in wild-type cells and the Δ*cas1-* and Δ*cas3*-deletion strains. However, crRNAs were absent in the Δcas*1821* and Δcas*1822* strains. These results are consistent with Cas6f generating mature crRNAs, which are then bound by Cas1821 and Cas1822 to maintain a stable crRNA pool in the cell. Thus, the absence of interference activity for the Δcas*1821* and Δcas*1822* strains correlates with the absence of crRNAs in these cells.

Previous studies on the type I-E CRISPR-Cas system in *E. coli* revealed that the heat-stable nucleoid-structuring (H-NS) protein, a global transcriptional repressor, can regulate the expression of CRISPR-Cas systems (49). We constructed a *S. putrefaciens* CN-32 Δ*hns* strain and observed slightly increased interference activity (Figure 3B). A two-fold increase in *cas* gene transcripts was detected via RT-qPCR (Supplementary Figure S2), which could explain increased interference.

### The minimal subtype I-F variant CRISPR-Cas system recognizes PAM sequences and targets both DNA strands

**Figure 4.**
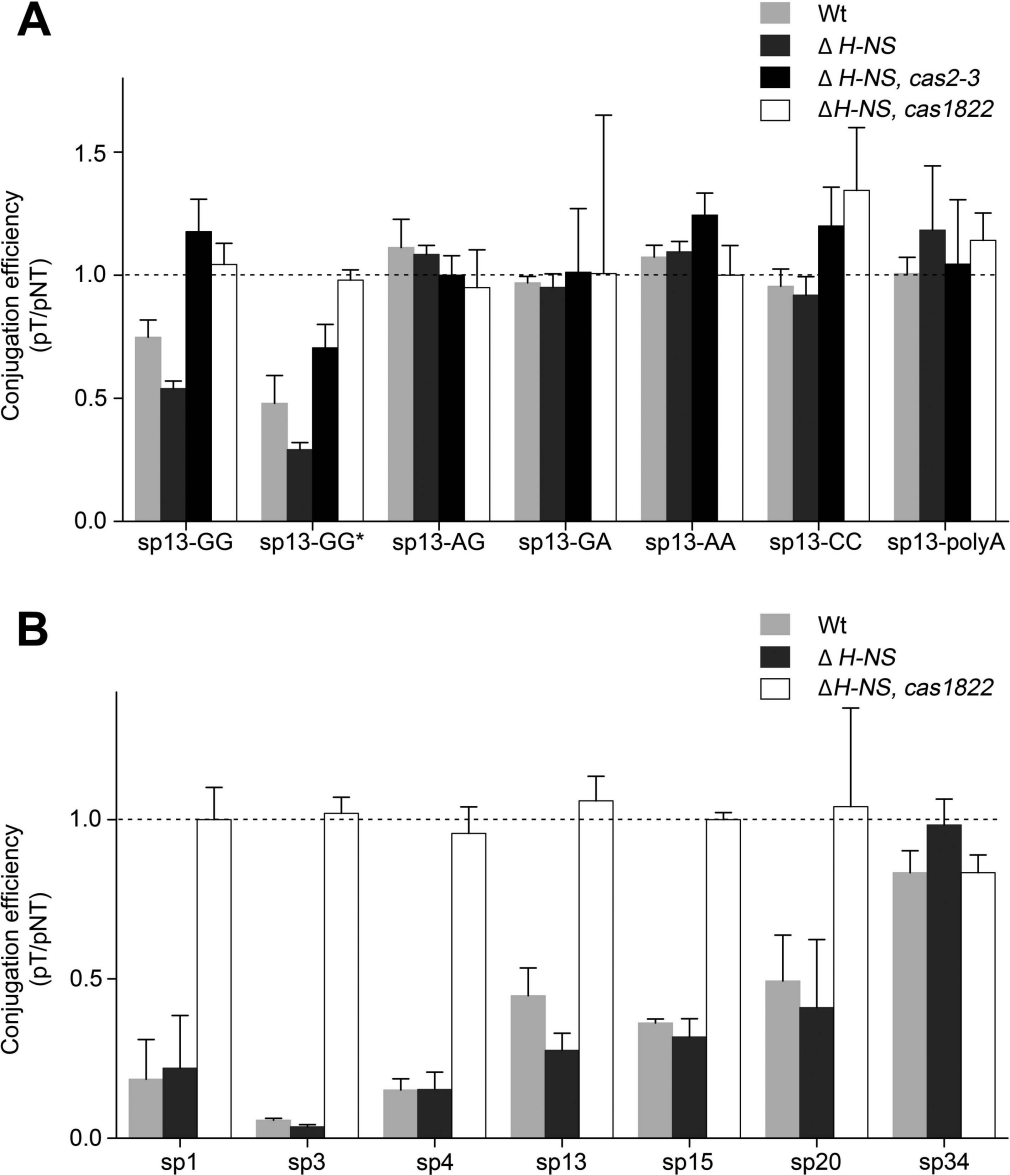
Analysis of the DNA target specificity of the *S. putrefaciens* CN-32 CRISPR–Cas system. Conjugation assays (see Figure 3) were used to analyze plasmid DNA target variants for DNA interference in *S. putrefaciens* CN-32 cells. (**A**) Interference was observed if the transferred DNA strand contained a sequence complementary to the crRNA13 (sp13-GG) or a sequence identical with spacer13 (sp13-GG*). Interference was evident for the wild-type and Δ*H-NS* strains, which contained all *cas* genes. However, the deletion of *cas3* (Sputcn32_1820) or cas*1822* abolished interference activity. The presence of the PAM sequence GG at the 3′ end of the DNA target (5′-protospacer-PAM-3′) was essential for the interference activity and PAM mutants (sp13-AG, sp13-GA, sp13-AA or sp13-CC) and a 10 nt poly-A sequence disrupting crRNA/DNA complementarity (sp13-polyA) were not targeted. (**B**) DNA interference activity was dependent on spacer sequence or crRNA abundance (spacers 1,3,4,13,15,20,34, see Figure 2).

Conjugation between the donor and the recipient cell requires the transfer of one strand of plasmid DNA through an intercellular cytoplasmic bridge, beginning at the origin of transfer and progressing in 5′ to 3′ direction. The transferred strand is converted into circular double-stranded plasmid DNA in the recipient and a new strand is synthesized in the donor to replace the transferred strand. In our experimental setup, the transferred strand can be directly targeted by the crRNA as it contains the sequence complementary to spacer13. To address if the minimal I-F variant system targets both DNA strands, a plasmid was constructed with the sequence of spacer13 on the transferred strand. Thus, the complementary target of crRNA 13 exists only after synthesis of the second strand in the recipient cell. The relative conjugation efficiency (pT/pNT) for this plasmid in both wild-type and Δ*H-NS* strains was comparable to the previous construct (Figure 4). This suggests that the I-F variant CRISPR–Cas system targets dsDNA.

Next, we aimed to investigate if the absence of a large subunit has an effect on the recognition of PAM sequences or the necessity for perfect crRNA/target complementarity. Variants of the targeted plasmid were constructed that contained PAM sequence mutations. These variants contained the dinucleotides AG, GA, AA or CC at the 3′ end of the spacer13 match on the target strand. In addition, a plasmid was constructed with the PAM-adjacent 10 nt of the protospacer (including the seed sequence) being replaced by a poly-A stretch. The relative conjugation efficiency (pT/pNT) of these constructs highlighted a loss of interference activity and demonstrated that PAM sequences are recognized in *S. putrefaciens* CN-32 (Figure 4A).

The RNA-Seq analysis of crRNA production revealed a highly variable crRNA abundance profile (Figure 2). We investigated if the abundance of individual crRNAs is correlated with the interference activity. Thus, we generated targeted plasmids with GG PAM sequences in which the spacer13 target was replaced with spacer1, spacer3 (abundant crRNAs) or spacer4, spacer15, spacer20 and spacer34 targets (low crRNA abundance). We performed conjugation assays and verified that highly abundant crRNAs (spacers1,3,13) yield efficient interference activity (Figure 4B). In agreement, the observed absence of stable crRNAs with spacer34 in *S. putrefaciens* CN-32 correlated with a complete loss of interference activity. However, interference activity was observed for crRNAs with spacer4, spacer15 and spacer20 even though only minimal amounts of these crRNAs were detected in our RNA-Seq analysis (Figure 4B). In general, crRNAs with promoter-adjacent spacers showed the highest interference activity. These results indicate that variable crRNA abundance is a factor that can potentially influence the efficiency of DNA targeting.

### Characterization of recombinant Cas1821 as a helical Cascade backbone protein

**Figure 5.**
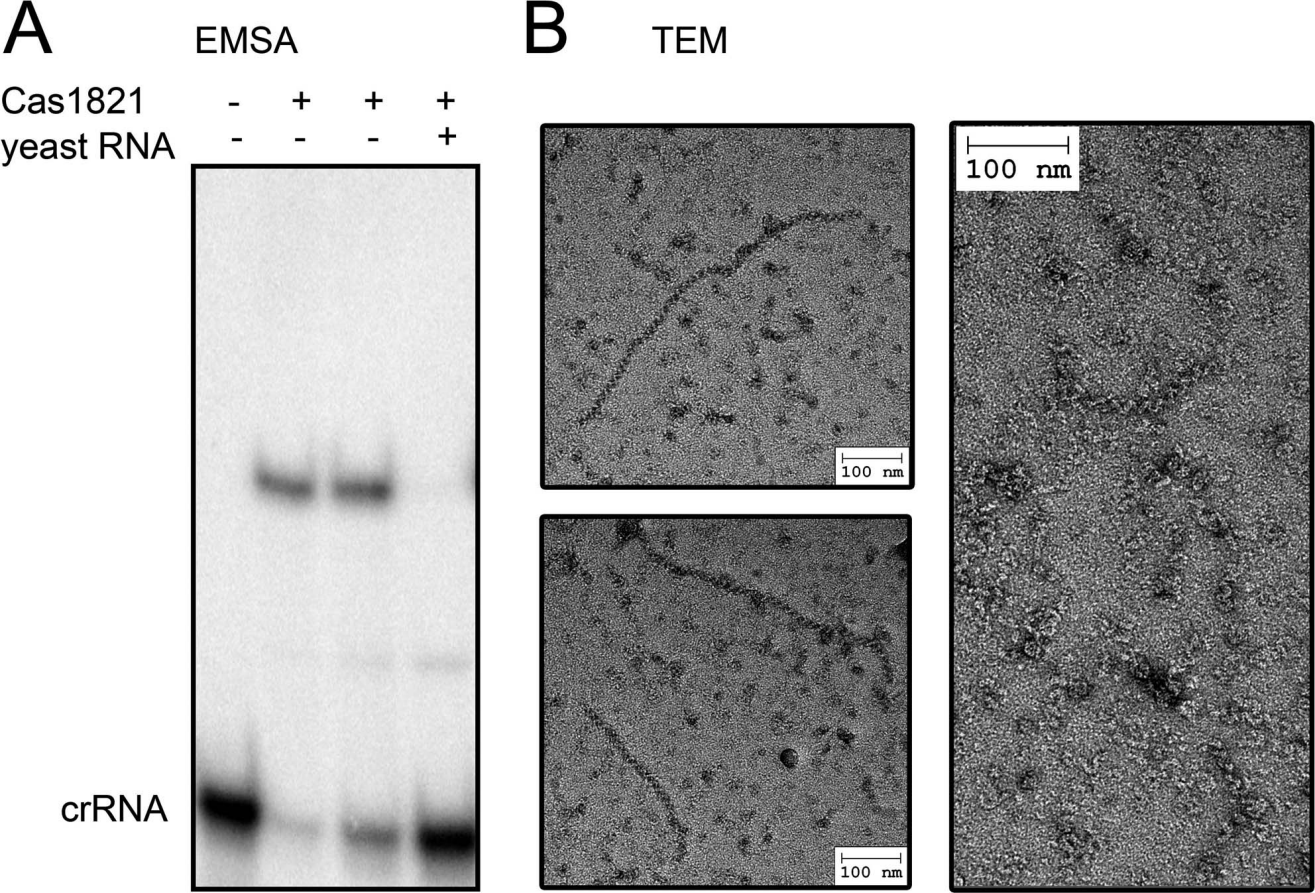
Characterization of recombinant Cas1821 protein. (**A**) EMSA assays indicate the binding of 5′-γ[^32^P]-ATP labeled crRNA by 250 nM Cas1821 (lane 2) or 500 nM Cas1821 (lane 3). Bands were separated by 6% native PAGE. The RNA binding is unspecific and outcompeted by yeast total RNA. (**B**) Transmission electron micrographs of negatively stained helical structures formed by RNA-bound recombinant Cas1821.

The absence of observable crRNAs in the Δ*cas1821* and Δ*cas1822* strains suggests that the encoded Cas proteins are required for maintaining a stable crRNA pool in the cell. Thus, these two proteins, Cas1821 and Cas1822, could fulfill the roles of Cas7 and Cas5 proteins even though sequence similarity calculations did not indicate them as members of these Cas protein families (26). We attempted to individually produce recombinant *S. putrefaciens* CN-32 Cas1821 and Cas1822 proteins in *E. coli*. The production of Cas1822 did not yield soluble protein in various expression conditions. However, soluble recombinant Cas1821 was produced as a SUMO-tag fusion construct. The SUMO-Cas1821 was purified via Ni-NTA chromatography and co-eluted with bound nucleic acid contaminants. Therefore, a high-salt washing step with a buffer including 1 M NaCl was added before protein elution from the Ni-NTA column, and a second cation exchange chromatography step was included in the purification protocol. This procedure yielded Cas1821 with a purity of >95% and without nucleic acid contaminants. Gel-elution chromatography of Cas1821 revealed a ∼35 kDa monomer (Supplementary Figure S3). Electrophoretic mobility shift assays of Cas1821 with radiolabeled crRNA transcripts revealed a slow migrating band indicating the crRNA-binding potential of Cas1821 (Figure 5A). This band disappeared upon addition of 1 μg of competitor yeast RNA, suggesting a non-specific interaction. This behavior is in agreement with the observed non-specific binding of *E. coli* contaminant nucleic acids. Recombinant Cas1821 bound to these contaminants eluted near the void volume during gel-elution chromatography (Supplementary Figure S3). These fractions were visualized by electron microscopy and revealed the formation of long helical filament-structures of varying length (Figure 5B). These observations suggest that Cas1821 could fulfil the role of Cas7 which was shown to form the helical Cascade backbone filament after unspecific RNA binding in other CRISPR-Cas subtypes (e.g. Type I-A, Type I-E).

### *In vivo* characterization of potential DNA target interaction partners

The absence of a large subunit implies that other Cas proteins can substitute for its role(s) during DNA target interaction. However, even for previously investigated and related subtype I-F CRISPR–Cas systems, the role of the large subunit (Csy1) is not clear, and it is not known which Cas protein is responsible for PAM recognition (44). The *S. putrefaciens* CN-32 CRISPR-Cas system is flanked by a homolog of the *DinG* gene (*Sputcn32_1824*), an ATP-dependent DNA helicase that was predicted to be a functional component of Type I-U CRISPR-Cas systems (26). To test if this protein plays a role in DNA interference, we constructed a Δ*DinG* strain. We did not observe a loss of interference activity in our conjugation assays indicating that this DinG homolog does not promote DNA interference and is not a component of subtype I-F variant Cascade (Supplementary Figure S4). This is in agreement with the observation that this DinG homolog is conserved in *S. putrefaciens* strains without CRISPR-arrays or other CRISPR-Cas subtypes.

**Figure 6.**
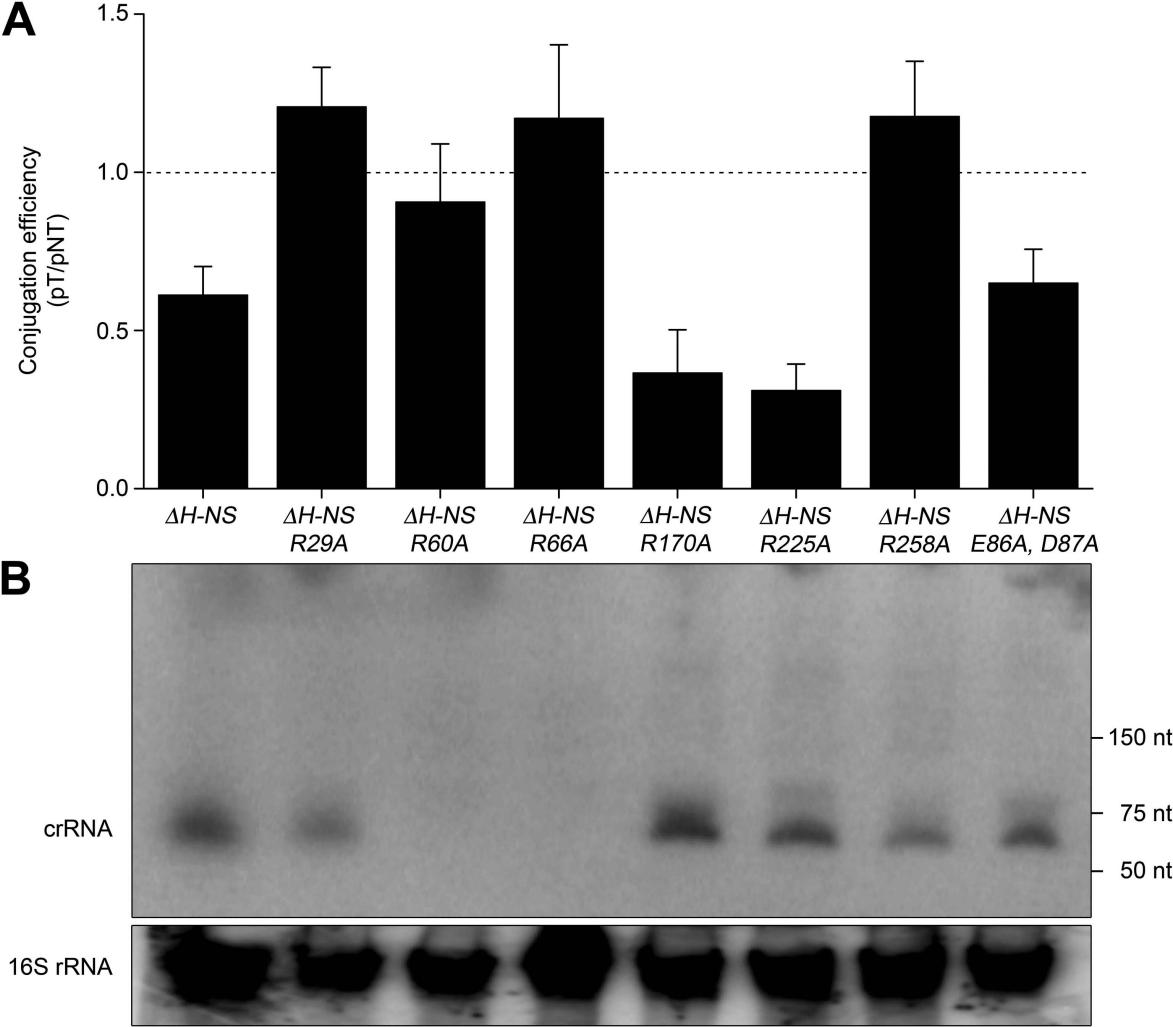
*In vivo* characterization of Cas1822 point-mutants. (**A**) *S. putrefaciens* CN-32 strains carrying Cas1822 proteins with the indicated point mutations were tested for DNA interference using the conjugation assay (see Figure 3). (**B**) Northern Blot analyses with anti-repeat probes revealed the cellular crRNA pool of these strains.

It is possible that Cas1822 interacts with the DNA target, as arginine residues are known to mediate DNA recognition in Cas proteins (50). A multiple alignment of Cas1822 proteins revealed conserved basic amino acids and a potential Asp/Glu motif (Supplementary Figure S5). Therefore, we assayed if mutations of conserved arginine residues in Cas1822 have an effect on interference activity. We constructed point mutations of the *cas1822* gene at the appropriate positions to generate corresponding Ala substitutions in Cas1822 *in vivo*, and these variants were tested for interference activity. Interference was observed for Δ*hns/R170A*, Δ*hns/R225A* and Δ*hns/E86A,D87A* strains (Figure 6A). A Northern blot analysis verified the presence of mature crRNAs in these strains (Figure 6B). In contrast, the two strains Δ*hns/R60A* and Δ*hns/R66A* lacked interference activity and stable crRNAs. We propose that these arginine residues are required for Cascade formation and crRNA protection. Finally, the two strains Δ*hns/R29A* and Δ*hns/R258A* did not show interference activity even though a stable crRNA pool was observed in the cell. Thus, these arginine residues are possibly involved in either the direct interaction with target DNA or with the Cas component that recognizes the PAM motif. Both scenarios are discussed below.

### Production of recombinant subtype I-F variant Cascade

**Figure 7.**
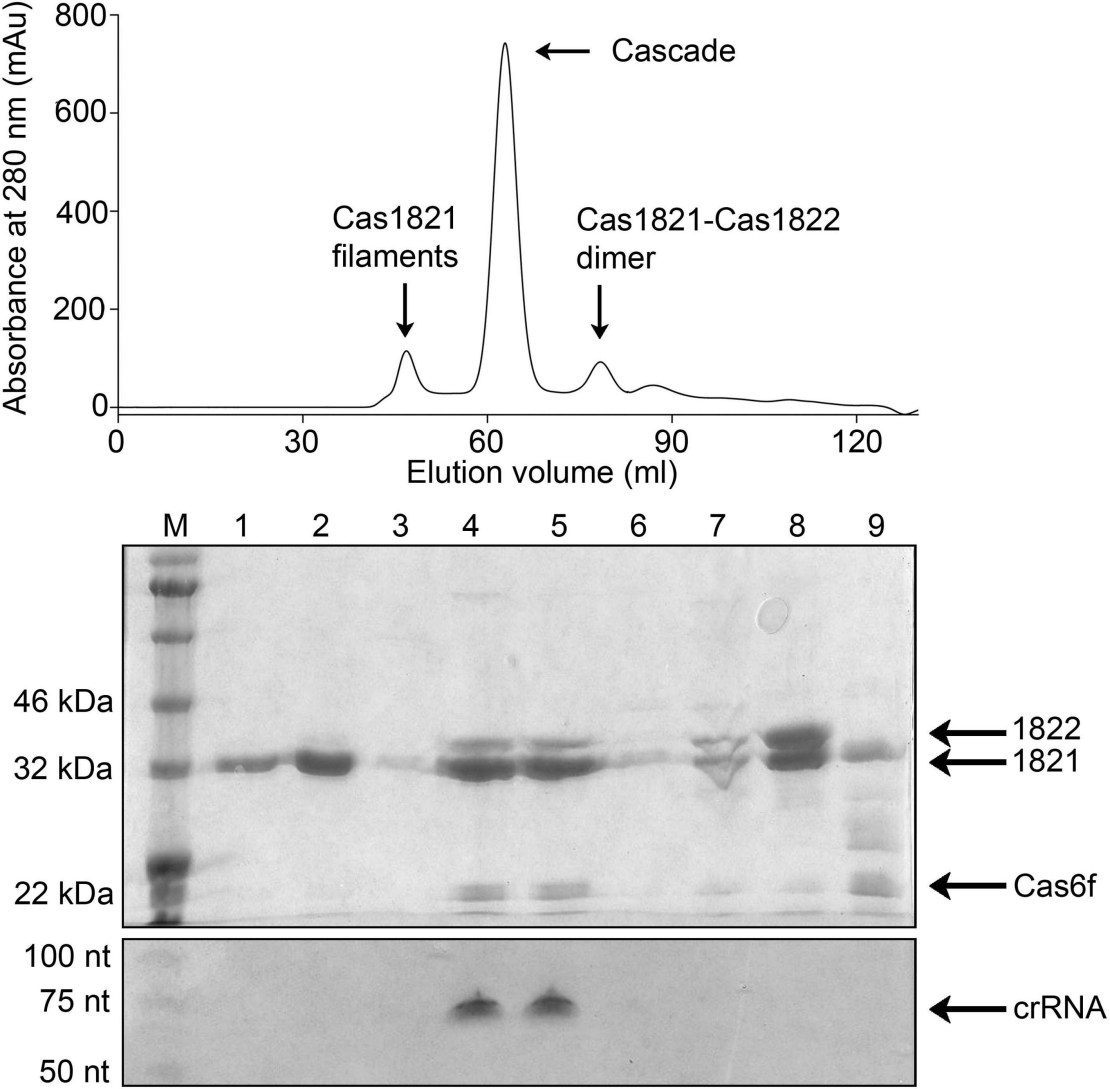
Co-purification of a recombinant subtype I-F variant Cascade complex. His-tagged Cas1821 co-eluted with Cas1822, Cas6f and mature crRNA during gel-elution chromatography (fractions 4 and 5) which verified Cascade complex formation. Cas1821 filaments were observed in the void volume (fractions 1 and 2). Dimers of Cas1821 and Cas1822 (fraction 8) were identified. SDS-PAGE (middle) and 8M urea PAGE (bottom) was used to separate the protein and RNA content of the fraction indicated in the gel-elution chromatogram (top).

Finally, we aimed to verify that Cas1821 and Cas1822 are assembled into a Cascade complex. Thus, we transformed two plasmids into *E. coli* to facilitate the T7 RNA polymerase-driven production of (i) a pre-crRNA transcript and (ii) the Cas proteins Cas1821, Cas1822 and Cas6f. Only Cas1821 contains a His-Tag, which was successfully used to purify a Cascade ribonucleoprotein complex from *E. coli*. This complex was subjected to gel elution chromatography and the RNA and protein content of the fractions was analyzed (Figure 7). A distinct peak revealed the formation of a stable complex with an estimated size of approximately 215 kDa. The Cas1821 protein was overrepresented in the complex which supports its role as Cascade backbone. Cas1822 and Cas6f were co-purified and are suggested to be the proteins that cap the 3′ hairpin tag (Cas6f) and the 8 nt 5′ tag (Cas1822) of the crRNA. Indeed, mature crRNA bands were co-purified in the Cascade fraction. This verified that Cas6f processed the pre-crRNA transcript in *E. coli* and likely loaded the Cascade complex. In addition, Cas1821-Cas1822 dimers were detected which suggests that these units are similar to the Cas5–Cas7 dimers observed for the type I-A Cascade.

## DISCUSSION

The Cas protein content of CRISPR-Cas systems varies between different subtypes. The Type I systems target DNA and are characterized by a multi-protein DNA interference complex termed Cascade. We analyzed the interference activity of the *S. putrefaciens* CN-32 CRISPR-Cas system, which contains a minimal Type I set of five Cas proteins. Three of these proteins can be classified into subtype I-F protein families. One of these proteins is Cas6, the endonuclease responsible for pre-crRNA cleavage reaction. Cas6 variants of the different CRISPR-Cas subtypes show different RNA recognition and cleavage features. The *S. putrefaciens* Cas6 enzyme is a Cas6f variant. A catalytic histidine residues at position 29 was found to be essential for cleavage activity, similar to the well-characterized Cas6f of *P. aeruginosa* (18). The mature crRNAs contain a standard 8 nt 5′ tag and a stable hairpin formed by the repeat sequence at the 3′ end, which prevents further 3′-terminal trimming. The 3′-terminal hairpin structures have also been found in other subtypes and are e.g. described for crRNAs found in *E. coli* and *P. aeruginosa* (6,18). In these systems, the corresponding Cas6 enzymes remain stably associated with the hairpin after cleavage and protect the crRNA from further degradation (18,37,51). This stable interaction of Cas6f to the crRNA is suggested to serve as a starting point for the assembly of the Cascade backbone by several Cas7 copies (17,23,24). This is suggested to allow Cas5 to bind and cap the 5′ tag of the crRNA. Thus, these three proteins bind and protect crRNA in Cascade and guarantee their stability and their ability to form base pairs with target DNA.

We showed that the deletion of Cas1821 or Cas1822 abolished interference activity in conjugation assays, which corresponded with the loss of an observable cellular crRNA pool. Analyses of *E. coli* and *Haloferax volcanii* Cascade structures and *Pseudomonas aeruginosa* Type I-F system activity showed that Cas5 and Cas7 proteins are essential for crRNA maintenance and stability (52–54). Thus, we propose that Cas1821 and Cas1822 can fulfill the roles of these two proteins. Accordingly, recombinant Cas1821, Cas1822 and Cas6f could be co-purified in Cascade assemblies with mature crRNAs. The non-specific RNA binding of Cas1821 led to the formation of oligomeric structures that could be visualized as helical filaments using electron microscopy. The formation of helical filaments was previously reported for Cas7 from *Thermoproteus tenax*, a sub-complex of Cas7 and Cas5a subunits from *Sulfolobus solfataricus* and for Type I-E Cascade in *E. coli* (22,39). Thus, we conclude that Cas1821 proteins build the helical backbone of the Cascade structures and serve as Cas7 proteins. The observed formation of Cas1821 and Cas1822 dimers suggest that these could be the initial building blocks of Cascade, similar to the Cas7 and Cas5a subunit dimers found in *S. solfataricus*.

Our RNA-Seq analyses indicated highly variable crRNA abundance patterns. The general crRNA abundance was lower than found for other subtypes, e.g. subtype I-B (*M. maripaludis)* or subtype I-A (*T. tenax*) (16,22). Usually, the most abundant crRNAs are the ones closest to the promoter in the CRISPR leader regions (16,22,55). These crRNAs contain the most recently acquired spacers. The variability of crRNA abundance could be explained by the presence of anti-crRNA sequences and internal promoters, which were observed in other cases (56). These scenarios are not apparent for the *S. putrefaciens* CRISPR array. It is plausible that the Cascade loading efficiency differs for different crRNAs. Additionally, pre-crRNA could form internal structures that would influence Cas6f cleavage site availability.

The assembly of crRNAs into Cascade complexes containing multiple Cas1821 copies, Cas1822 and Cas6f highlights the universality of this architecture in Type I systems. It is apparent that small and large subunits are missing in this subtype I-F variant CRISPR-Cas system. We utilized a conjugation assay to study if the loss of these subunits has an influence on known features of Type I-mediated DNA interference. However, we observed that this system targets dsDNA and depends on the presence of a PAM sequence as expected for a Type I CRISPR-Cas activity. The PAM sequence was found to be GG and mutations in this sequence resulted in the loss of interference activity. The PAM sequence is shared with subtype I-F CRISPR-Cas systems (14,57). We asked if undetected Cas proteins exist in the genomes of organisms that contain the subtype I-F variant, which could fulfill the function of small and large subunits. It should be noted that the small subunit is also missing for subtype I-F CRISPR-Cas systems. The subtype I-F variant CRISPR-Cas system was identified in nine mesophilic beta- and gamma-proteobacteria: *S. putrefaciens* (strains CN-32 & 200), *Oligella ureolytica* (strain DSM 18253), *Pseudoalteromonas tunicata* (strain D2), *Legionella pneumophila* (strain 2300/99 Alcoy), *Oxalobacter formigenes* (strain OXCC13), *P. profundum* (strain SS9), *Vibrio cholerae* (strain TM 11079–80) and *Methylophaga nitratireducenticrescens*. Phylogenetic profiling did not reveal additional Cas proteins that co-evolved with the unusual Cascade assemblies. A homolog of a DinG helicase was identified downstream of the CRISPR array, which was reported to be part of Type I-U CRISPR-Cas system (26). However, these enzymes are also found in related *Shewanella* strains without CRISPR systems or with different CRISPR-Cas subtypes. A deletion of the *dinG* gene did not abolish the interference activity indicating that the encoded protein is not required for subtype I-F variant Cascade activity. In conclusion, we do not see any evidence for additional Cascade components that are not encoded in the minimal cas gene operon. To date, RNase III and PNPase are the only non-Cas proteins that can be involved in conferring CRISPR-Cas based immunity (58,59). Both proteins play a key role in crRNA generation and are not involved in DNA interactions. The large subunit of the subtype I-E *E. coli* Cascade has been shown to interact with the PAM motif and to recruit Cas3. In addition, both small and large subunits are required for the stabilization of the crRNA-target hybrid (23,60). The role of the large subunit (Csy1) of subtype I-F CRISPR-Cas systems is not fully understood yet. We hypothesize that either Cas1822 and/or Cas3 are involved in DNA target PAM recognition. We showed that the mutation of conserved arginine residues in Cas1822 influenced DNA interference activity even in the presence of a stable cellular crRNA pool. Cas3 interacts with the DNA and could provide additional DNA target selectivity. It is interesting to note that the subtype I-F and subtype I-F variant Cas3 enzymes contain an N-terminal portion that resembles Cas2 (44). Only this region is conserved between Cas3 enzymes found in different *Shewanella* strains, while the larger C-terminal portion shows no apparent homology. Thus, unexpected Cas3 functions could be encoded in this part. The soluble recombinant production of *S. putrefaciens* Cas1822 and Cas3 was not successful and we rely on future studies of macromolecular Cascade structures to pinpoint the molecular details of PAM recognition mechanisms in subtype I-F and subtype I-F variant CRISPR-Cas systems. The loss of large and small subunits might coincide with the evolution of specialized minimal Type I Cascade systems that still rely on PAM motif recognition for DNA target specificity.

## Supplementary Material

SUPPLEMENTARY DATA
