# Supplementary material for: Interference activity of a minimal Type I CRISPR–Cas system from Shewanella putrefaciens
Source: Nucleic Acids Res. 2015 Oct 10;43(18):8913–23. doi: 10.1093/nar/gkv882 (PMC4605320; doi:10.1093/nar/gkv882)
Supplement: SUPPLEMENTARY DATA [file supp_43_18_8913__index.html]

Interference activity of a minimal Type I CRISPR–Cas system from Shewanella putrefaciens — SUPPLEMENTARY DATA 

# Interference activity of a minimal Type I CRISPR–Cas system from *Shewanella putrefaciens*

## SUPPLEMENTARY DATA

- SUPPLEMENTARY DATA
- SUPPLEMENTARY DATA
