# Supplementary material for: Interference activity of a minimal Type I CRISPR–Cas system from Shewanella putrefaciens
Source: Nucleic Acids Res. 2015 Oct 10;43(18):8913–23. doi: 10.1093/nar/gkv882 (PMC4605320; doi:10.1093/nar/gkv882)
Supplement: SUPPLEMENTARY DATA [file supp_gkv882_nar-01357-h-2015-File009.docx]

**SUPPLEMENTARY DATA**

**Interference activity of a minimal Type I CRISPR-Cas system from *Shewanella putrefaciens***

Srivatsa Dwarakanath, Susanne Brenzinger, Daniel Gleditzsch, André Plagens, Andreas Klingl, Kai Thormann and Lennart Randau

**Supplementary Tables**

Supplementary Table S1: List of Strains and Plasmids used.

| **Strain** | **Description** | **Reference** |
| --- | --- | --- |
| *Escherichia coli* DH5α | FhuA2 Δ(argF-lacZ)U169 phoA glnV44 Φ80 Δ(lacZ) M15 gyrA96 recA1 relA1 endA1 thi-1 hsdR17 | Invitrogen |
| *E. coli* DH5α λpir | sup E44, ΔlacU169 (ΦlacZΔM15), recA1, endA1, hsdR17, thi-1, gyrA96, relA1, λpir phage lysogen | Miller and Mekalanos, 1988 (1) |
| *E. coli* BL21(DE3) pLysS | F-ompT hsdSB(rB- mB-) gal dcm (DE3) pLysS (Spec^R^) | Invitrogen |
| *E. coli* WM3064 | thrB1004 pro thi rpsL hsdS lacZΔM15 RP4-1360 Δ(araBAD)567 ΔdapA1341::[erm pir] | W. Metcalf, University of Illinois, Urbana |
| *Shewanella putrefaciens* CN-32 |  | Fredrickson et al., 1998 (2) |
| *S. putrefaciens* CN-32 Δ*1819* | 1819 (cas1) knockout | This study |
| *S. putrefaciens* CN-32 Δ*1820* | *1820 (cas2-3)* knockout | This study |
| *S. putrefaciens* CN-32 Δ*1821* | *1821* knockout | This study |
| *S. putrefaciens* CN-32 Δ*1822* | *1822* knockout | This study |
| *S. putrefaciens* CN-32 Δ*2512* | *2512 (H-NS)* knockout | This study |
| *S. putrefaciens* CN-32 Δ*1824* | *1824 (DinG)* knockout | This study |
| *S. putrefaciens* CN-32 Δ*2512*, Δ*1820* | *H-NS* and *cas2-cas3* double knockout | This study |
| *S. putrefaciens* CN-32 Δ*2512*, Δ*1822* | *H-NS* and *1822* double knockout | This study |
| *S. putrefaciens* CN-32 Δ*2512*, Δ*1824* | *H-NS* and *DinG* double knockout | This study |
| *S. putrefaciens* CN-32 Δ*2512*, *1822(R29A)* | *H-NS* knockout strain with a R29A mutation in *1822* gene product | This study |
| *S. putrefaciens* CN-32 Δ*2512*, *1822(R60A)* | *H-NS* knockout strain with a R60A mutation in *1822* gene product | This study |
| *S. putrefaciens* CN-32 Δ*2512*, *1822(R66A)* | *H-NS* knockout strain with a R66A mutation in *1822* gene product | This study |
| *S. putrefaciens* CN-32 Δ*2512*, *1822(R170A)* | *H-NS* knockout strain with a R170A mutation in *1822* gene product | This study |
| *S. putrefaciens* CN-32 Δ*2512*, *1822(R225A)* | *H-NS* knockout strain with a R225A mutation in *1822* gene product | This study |
| *S. putrefaciens* CN-32 Δ*2512*, *1822(R258A)* | *H-NS* knockout strain with a R258A mutation in *1822* gene product | This study |
| *S. putrefaciens* CN-32 Δ*2512*, *1822(E86A,D87A)* | *H-NS* knockout strain with a E86A and D87A mutations in *1822* gene product | This study |
| *S. putrefaciens* CN-32 *1820(H156A,D157A)* | Wildtype strain with a H156A and D157A mutations in *1820* gene product | This study |

| **Plasmid** | **Description** | **Source, Reference** |
| --- | --- | --- |
| pET20b | T7 polymerase based expression vector, Amp^R^ | Novagen |
| pNPTS138-R6KT | *mobRP4*+ *ori*-R6K *sacB*; suicide plasmid for in-frame deletions, Kan^R^ | Lassak et al., 2010 (3) |
| pUC19 | cloning vector, Amp^R^ | New England Biolabs |
| pBBR1MCS2 | Kan^R^; *lacZ* P*_lac_* P*_T7_ rep* | Kovach et al., 1995 (4) |
| pNPTS138-R6KT-Δ*cas1* | *cas1* deletion fragment in pNPTS138-R6KT | This study |
| pNPTS138-R6KT-Δ*cas2-cas3* | *cas2-cas3* deletion fragment in pNPTS138-R6KT | This study |
| pNPTS138-R6KT-Δ*1821* | *1821* deletion fragment in pNPTS138-R6KT | This study |
| pNPTS138-R6KT-Δ*1822* | *1822* deletion fragment in pNPTS138-R6KT | This study |
| pNPTS138-R6KT-Δ*H-NS* | *H-NS* deletion fragment in pNPTS138-R6KT | This study |
| pNPTS138-R6KT-Δ*DinG* | *DinG* truncation fragment in pNPTS138-R6KT | This study |
| pNPTS138-R6KT-1822-R29A | fragment carrying R29A mutation of *1822* in pNPTS138-R6KT | This study |
| pNPTS138-R6KT-1822-R60A | fragment carrying R60A mutation of *1822* in pNPTS138-R6KT | This study |
| pNPTS138-R6KT-1822-R66A | fragment carrying R66A mutation of *1822* in pNPTS138-R6KT | This study |
| pNPTS138-R6KT-1822-R170A | fragment carrying R170A mutation of *1822* in pNPTS138-R6KT | This study |
| pNPTS138-R6KT-1822-R225A | fragment carrying R225A mutation of *1822* in pNPTS138-R6KT | This study |
| pNPTS138-R6KT-1822-R258A | fragment carrying R258A mutation of *1822* in pNPTS138-R6KT | This study |
| pNPTS138-R6KT-1822-E86A,D87A | fragment carrying E86A and D87A mutations of *1822* in pNPTS138-R6KT | This study |
| pNPTS138-R6KT-Cas2-3-H156A,D157A | fragment carrying H156A and D157A mutations of *Cas2-3* in pNPTS138-R6KT | This study |
| pET20b-Cas6f | Cas6f with a 6x His-tag (C-terminal) | This study |
| pEC-1821 | 1821 with an N-terminal 6x His-tag and SUMO-Tag | This study |
| pRSF567 | pRSFduet1 carrying 1821, 1822 and Cas6 as an operon with an N-terminal 6x his-tag on 1821 | This study |
| pUCcrRNA4 | pUC19 carrying a repeat-spacer4-repeat (partial) fragment | This study |
| pUCsp3-rep-sp4 | pUC19 carrying a spacer3-repeat-spacer4 fragment | This study |
| pBBR1MCS2-sp1-GG | pBBR1MCS2 containing *S. putrefaciens* CN-32 spacer 1 with PAM ´GG´ on the 3´end of the non-target strand | This study |
| pBBR1MCS2-sp3-GG | pBBR1MCS2 containing *S. putrefaciens* CN-32 spacer 3 with PAM ´GG´ on the 3´end of the non-target strand | This study |
| pBBR1MCS2-sp4-GG | pBBR1MCS2 containing *S. putrefaciens* CN-32 spacer 4 with PAM ´GG´ on the 3´end of the non-target strand | This study |
| pBBR1MCS2-sp13-GG | pBBR1MCS2 containing *S. putrefaciens* CN-32 spacer 13 with PAM ´GG´ on the 3´end of the non-target strand | This study |
| pBBR1MCS2-sp15-GG | pBBR1MCS2 containing *S. putrefaciens* CN-32 spacer 15 with PAM ´GG´ on the 3´end of the non-target strand | This study |
| pBBR1MCS2-sp20-GG | pBBR1MCS2 containing *S. putrefaciens* CN-32 spacer 20 with PAM ´GG´ on the 3´end of the non-target strand | This study |
| pBBR1MCS2-sp34-GG | pBBR1MCS2 containing *S. putrefaciens* CN-32 spacer34 with PAM ´GG´ on the 3´end of the non-target strand | This study |
| pBBR1MCS2-sp13rev-GG | pBBR1MCS2 containing *S. putrefaciens* CN-32 spacer 13 in the opposite orientation than before | This study |
| pBBR1MCS2-sp13-AA | pBBR1MCS2 containing *S. putrefaciens* CN-32 spacer 13 with PAM ´AA´ on the 3´end of the non-target strand | This study |
| pBBR1MCS2-sp13-GA | pBBR1MCS2 containing *S. putrefaciens* CN-32 spacer 13 with PAM ´GA´ on the 3´end of the non-target strand | This study |
| pBBR1MCS2-sp13-AG | pBBR1MCS2 containing *S. putrefaciens* CN-32 spacer 13 with PAM ´AG´ on the 3´end of the non-target strand | This study |
| pBBR1MCS2-sp13-CC | pBBR1MCS2 containing *S. putrefaciens* CN-32 spacer 13 with PAM ´CC´ on the 3´end of the non-target strand | This study |
| pBBR1MCS2-sp13-polyA | pBBR1MCS2 containing *poly A* in place of first 10 nucleotides of *S. putrefaciens* CN-32 spacer 13 | This study |

Supplementary Table 2. List of primers.

| **Name** | **Sequence 5 ’🡪 3’** |
| --- | --- |
| In frame deletion of Cas1 |  |
| Cas1 KO up fw | AGAATTCCCAGCAAACATCCATTGCCCAA |
| Cas1 KO up OL rv | TAATCCTTGAAAATCATCCATAACTCACCTCAGTA |
| Cas1 KO dwn OL fw | GATGATTTTCAAGGATTATAAGGTACAGAAGCTAT |
| Cas1 KO dwn rv | TCTGGGCCCATCTTCGGCTATGAAGTCCCTC |
| Cas1 check fw | AGACTGCGACCCATACTCGC |
| Cas1 check rv | TAACAGCGACACTTCATTATGCC |
| In frame deletion of Cas3 |  |
| Cas3 KO up fw | AGAATTCGCCGCGCTCGAGACGTT |
| Cas3 KO up OL rv | TAGATTTTCCATCATAGCTTCTGTACCTTATAATC |
| Cas3 KO dwn OL fw | GCTATGATGGAATAAATCATGCAAAAAGTAAACGGG |
| Cas3 KO dwn rv | TCTGGGCCCCATAGGAGATGTATTCTGTATCCCC |
| Cas3 check fw | AAAACGATTACTCGCCGCGA |
| Cas3 check rv | CTCTATTAGTATTGAACAGTTGCAGT |
| In frame deletion of Cas1821 |  |
| 1821 KO up fw | AGAATTCGTAGATCCTATTACCGTTCCACCA |
| 1821 KO up OL rv | CTCCTATTTTTTTTGCATGATTTATTCCTCATCTTCA |
| 1821 KO dwn OL fw | ATGCAAAAAAAATAGGAGGTTGCATGAAAATAATCA |
| 1821 KO dwn rv | TCCGGGCCCTTAACGGGTTTAACTCAATAGATCCT |
| 1821 check fw | CACACTGAACGTAATCGAGGG |
| 1821 check rv | CTGATTAAGGCTCTCCAAGCG |
| In frame deletion of Cas1822 |  |
| 1822 KO up fw | AGAATTCGCAGGAGCAAGAGATAGTACATC |
| 1822 KO up OL rv | CTAAAGCTTGATTATTTTCATGCAACCTCCTATTT |
| 1822 KO dwn OL fw | AAAATAATCAAGCTTTAGGAGCAATGATGAACTC |
| 1822 KO dwn rv | GTGCTGCAGCAAGGGGGTGAGCTTGAATGT |
| 1822 check fw | TTGGAAGATTTTGTTGATCAATTGGG |
| 1822 check rv | TAAAACCAAGGTACTGTAGCGGT |
| In frame deletion of H-NS |  |
| H-NS KO up fw | CGAATTCATACTGACAACAACCACTTCGGTA |
| H-NS KO up OL rv | CCTTTTACTGCGAGTTTGTATGGAATCTATTTTCGTCCCACTTTAATTTTTAAT |
| H-NS KO dwn OL fw | ATTAAAAATTAAAGTGGGACGAAAATAGATTCCATACAAACTCGCAGTAAAAGG |
| H-NS KO dwn rv | GGTTGGATCCGGTAAAGGGATCGATAAATCCGGG |
| H-NS check fw | TGCAGTACGTAGCTGTTCTTGTA |
| H-NS check rv | TAATATCATAAACAGGGCGTGTCAGT |
| In frame truncation of DinG |  |
| DinG KO up fw | GCGGAATTCTATCTTAAATCAGCATAATGCTTCGAACTC |
| DinG KO up OL rv | TTGTCCGCCAATATTATTAACAAGATGTTTTCTTGCAGATTTTTACTGCCCGTACTAATG |
| DinG KO dwn OL fw | CATTAGTACGGGCAGTAAAAATCTGCAAGAAAACATCTTGTTAATAATATTGGCGGACAA |
| DinG KO dwn rv | CGCGGATCCACCCAACAATAAAGAAGGAAAATAATATGC |
| DinG check fw | GTAAATCTGAATAGCCGCACACGCGGCTTTAA |
| DinG check rv | ATACCTACATCATATTCACACAACATGCGC |
| *In vivo* mutation in Cas1822 |  |
| 1822 fw | GAGGAATTCGGAGCAAGAGATAGTACATCATTTTTTTCA |
| 1822 rv | AGCGGATCCTGAGCTTGAATGTCGCTAAACTCAAAGAAT |
| R29A fw | CATTGAGCCTAAAAATTCGGCGCCTTTTTTCGGTACAGGT |
| R29A rv | ACCTGTACCGAAAAAAGGCGCCGAATTTTTAGGCTCAATG |
| R60A fw | TTCGTTGGTCGCCAATTAAAGCATTTAGTACTCCCATTACGG |
| R60A rv | CCGTAATGGGAGTACTAAATGCTTTAATTGGCGACCAACGAA |
| R66A fw | CGCGCCTGATAAAGCTTTGCTTGGTCGCCAATTAAACG |
| R66A rv | CGTTTAATTGGCGACCAAGCAAAGCTTTATCAGGCGCG |
| R170A fw | GATTAAGGCTCTCCAAGGCATTGATAATACTTAACGGGTTTAACTCAATA |
| R170A rv | TATTGAGTTAAACCCGTTAAGTATTATCAATGCCTTGGAGAGCCTTAATC |
| R225A fw | TATTAAAGCTAGTTTCTAAGGCGGCAAGCTGAAGGTAAAGCG |
| R225A rv | CGCTTTACCTTCAGCTTGCCGCCTTAGAAACTAGCTTTAATA |
| R258A fw | TTTTTTGGGTCCCGTTGTATATGCGTCCATGAAGTCTTTTTTAGTG |
| R2258A rv | CACTAAAAAAGACTTCATGGACGCATATACAACGGGACCCAAAAAA |
| E86A D87A fw | GCTTATCTATAAAGCTGACTTTAGCTGCCAGTGCTTCGAAGTAATAAGCG |
| E86A D87A rv | CGCTTATTACTTCGAAGCACTGGCAGCTAAAGTCAGCTTTATAGATAAGC |
| In vivo mutation in Cas2-3 |  |
| Cas2-3 fw | GAGGAATTCATGATGGTGACATTCATCAGTCAGTGCGAG |
| Cas2-3 rv | CGGATATCGTTGTTTAGTGCATTGAGTTTCACATTGGT |
| H156A D157A fw  H156A D157A rv | AACTTTGGGTCAATTTTCCCTAAAGCAGCCAAGCAACCTGCAATAAAATTCGC  GCGAATTTTATTGCAGGTTGCTTGGCTGCTTTAGGGAAAATTGACCCAAAGTT |
| *In vivo* conjugation assay plasmids |  |
| sp1-GG-fw | AATTCCCTCAACCAAATCATAAATTGCGCGACCACATTGG |
| sp1-GG-rv | GATCCCAATGTGGTCGCGCAATTTATGATTTGGTTGAGGG |
| sp3-GG-fw | AATTCCCTATTGAGTTTGCATCAAGCACGGTATGGCGCTG |
| sp3-GG-rv | GATCCAGCGCCATACCGTGCTTGATGCAAACTCAATAGGG |
| sp4-GG-fw | AATTCCCTATCGCCCAGCAAGACGCGCAAACCTATAACCG |
| sp4-GG-rv | GATCCGGTTATAGGTTTGCGCGTCTTGCTGGGCGATAGGG |
| sp13-GG-fw | AATTCCCCGTTATGTCTGTTACACTGTCAACTTCCAATAG |
| sp13-GG-rv | GATCCTATTGGAAGTTGACAGTGTAACAGACATAACGGGG |
| sp15-GG-fw | AATTCCCGTTAACAGTTGCTGGCAACGTCTTTTGCACCTG |
| sp15-GG-rv | GATCCAGGTGCAAAAGACGTTGCCAGCAACTGTTAACGGG |
| sp20-GG-fw | AATTCCCGCAGGGACTCGCGCAAAGGCTGGCGTTATGAGG |
| sp20-GG-rv | GATCCCTCATAACGCCAGCCTTTGCGCGAGTCCCTGCGGG |
| sp34-GG-fw | AATTCCCTGTTGGATTTGGCTCTTGGTTCGGCGCTTCGGG |
| sp34-GG-rv | GATCCCCGAAGCGCCGAACCAAGAGCCAAATCCAACAGGG |
| sp13rev-GG-fw | AATTCTATTGGAAGTTGACAGTGTAACAGACATAACGGGG |
| sp13rev-GG-rv | GATCCCCCGTTATGTCTGTTACACTGTCAACTTCCAATAG |
| sp13-AA-fw | AATTCTTCGTTATGTCTGTTACACTGTCAACTTCCAATAG |
| sp13-AA-rv | GATCCTATTGGAAGTTGACAGTGTAACAGACATAACGAAG |
| sp13-GA-fw | AATTCTCCGTTATGTCTGTTACACTGTCAACTTCCAATAG |
| sp13-GA-rv | GATCCTATTGGAAGTTGACAGTGTAACAGACATAACGGAG |
| sp13-AG-fw | AATTCCTCGTTATGTCTGTTACACTGTCAACTTCCAATAG |
| sp13-AG-rv | GATCCTATTGGAAGTTGACAGTGTAACAGACATAACGAGG |
| sp13-CC-fw | AATTCGGCGTTATGTCTGTTACACTGTCAACTTCCAATAG |
| sp13-CC-rv | GATCCTATTGGAAGTTGACAGTGTAACAGACATAACGCCG |
| sp13-polyA-fw | AATTCCCAAAAAAAAAAGTTACACTGTCAACTTCCAATAG |
| sp13-polyA-rv | GATCCTATTGGAAGTTGACAGTGTAACTTTTTTTTTTGGG |
| pet20b-Cas6f-fw | TTTAGGACATATGATGAACTCATATATTGATA |
| pet20b-Cas6f-rv | AAGAACAAAAATAAACTCGAGAAACCAAGG |
| pUC-sp3:rep:sp4 fw | GGATCCTAATACGACTCACTATAGGGAGATATTGAGTTTGCATCAAGCA |
| pUC-sp3:rep:sp4 rv | AAGCTTGGTTATAGGTTTGCGCGTCTTGCTGGGCGATA |
| pEC-1821-fw | ACCAGGAACAAACCGGCGGCCGCTCGATGCAAAAAGTAACGGGAATTAAAAGTGT |
| pEC-1821-rv | GCAAAGCACCGGCCTCGTTACTATTTTGCATAAAAATACTGTGCAAATGGC |
| pRSF567-fw | AGCGGATCCGATGCAAAAAGTAACGGGAATTAAAAGTGTT |
| pRSF567-rv | GATTGCGGCCGCTTAAAACCAAGGTACTGTAGCGGTTTTGCT |
| pUC-crRNA4-fw | GATCCTAATACGACTCACTATAGGGAGAGTTCACCGCCGCACAGGCGGCTTAGAAATATCGCCCAGCAAGACGCGCAAACCTATAACCGTTCACCGCCGCACAGGCGGA |
| pUC-crRNA4-rv | AGCTTCCGCCTGTGCGGCGGTGAACGGTTATAGGTTTGCGCGTCTTGCTGGGCGATATTTCTAAGCCGCCTGTGCGGCGGTGAACTCTCCCTATAGTGAGTCGTATTAG |
| Northern blot primers |  |
| repeat sequence | GTCTTGCTGGGCGATATTTC |
| 16S control | GCTTTACGCCCAGTAATTCC |
| qPCR primers |  |
| 16S-fw | GCCTTATCTGTGACGTTACC |
| 16S-rv | GCTTTACGCCCAGTAATTCC |
| 1819-Cas1-fw | AAGACGCCATCGTATTACCC |
| 1819-Cas1-rv | GGTCGGTAAAGGCTTGTAAC |
| 1820-Cas2,3-fw | GGCATAGTGCCAGTGAATAG |
| 1820-Cas2,3-rv | GTGGCTGTAATTGGGTGTTG |
| 1821-fw | GGCGTAGTGAATTGGAATGG |
| 1821-rv | CCGGTCAAGTTTGTGTAGC |
| 1822-fw | TGGCAGTACGGATCAGAATG |
| 1822-rv | GTGAAATCGAGAGCCAACAC |
| 1823-Cas6f-fw | TATACGGCTGAAGCCTGATG |
| 1823-Cas6f-rv | GGCTATTGAGCGTCACTAAC |

Supplementary Table 3. Transconjugant counts of the conjugation assays

This is a separate Excel file that details the raw transconjugant counts observed for the conjugation assays.

**Supplementary Figures**

**Figure S1. Cas6f endonuclease activity.** Recombinant Cas6f endonuclease (wild type and H29A active site mutant) was incubated with 5′-γ[32P]-ATP labeled spacer3-repeat-spacer4 transcript RNA. Cleavage products are indicated.

**Figure S2. Analysis of cas gene transcript abundance.** qPCR analysis was performed to compare *cas* gene transcript abundance between *S. putrefaciens* wild type and *ΔH-NS* strains.

**Figure S3. Filament formation of Cas1821.** Size exclusion chromatograms of Cas1821 with and without bound RNA. The top panel shows the elution profile of apo-Cas1821 measured at 280 nm and 260 nm. A single major peak corresponding to a mass of ~35 kDa was observed. When bound to RNA, most of the protein was found to elute in the void volume (bottom panel).

**Figure S4. Influence of DinG on DNA interference activity.** A deletion strain of *DinG* was generated in *S. putrefaciens* CN-32 wild type and Δ*H-NS* strains and tested for interference using the conjugation assay. The interference-inactive *ΔH-NS*,Δ*Cas1822* strain served as control.

**Figure S5. Conservation of Cas1822 residues.** A multiple sequence alignment of Cas1822from *S. putrefaciens* CN-32 and its homologs identified conserved arginine residues and a partially conserved Asp/Glu motif (marked by an asterisk).

**References**

1. Miller, V.L. and Mekalanos, J.J. (1988) A novel suicide vector and its use in

construction of insertion mutations: osmoregulation of outer membrane proteins and

virulence determinants in Vibrio cholerae requires toxR. *Journal of bacteriology*, 170,

2575-2583.

2. Fredrickson, J.K., Zachara, J.M., Kennedy, D.W., Dong, H., Onstott, T.C., Hinman,

N.W. and Li, S.-m. (1998) Biogenic iron mineralization accompanying the

dissimilatory reduction of hydrous ferric oxide by a groundwater bacterium.

*Geochimica et Cosmochimica Acta*, 62, 3239-3257.

3. Lassak, J., Henche, A.-L., Binnenkade, L. and Thormann, K.M. (2010) ArcS, the

cognate sensor kinase in an atypical Arc system of Shewanella oneidensis MR-1.

*Applied and environmental microbiology*, 76, 3263-3274.

4. Kovach, M.E., Elzer, P.H., Hill, D.S., Robertson, G.T., Farris, M.A., Roop, R.M. and

Peterson, K.M. (1995) Four new derivatives of the broad-host-range cloning vector

pBBR1MCS, carrying different antibiotic-resistance cassettes. *Gene*, 166, 175-176.
